# Impact of Waste-HydroChar on the Rheological Behavior, Physical Properties, and Aging Resistance of Bitumen

**DOI:** 10.3390/ma19020245

**Published:** 2026-01-07

**Authors:** Nadka Tz. Dintcheva, Rosalia Teresi, Francesco Graziano, Giulia Infurna, Maurizio Volpe, Antonio Messineo, Clara Celauro

**Affiliations:** 1 Dipartimento di Ingegneria, Università degli Studi di Palermo, Viale delle Scienze Ed. 6, 90128 Palermo, Italy; rosalia.teresi@unipa.it (R.T.); francesco.graziano07@unipa.it (F.G.); giulia.infurna@unipa.it (G.I.); 2 Dipartimento di Ingegneria e Architettura, Università di Enna, Kore, Cittadella Universitaria, 94100 Enna, Italy; maurizio.volpe@unikore.it (M.V.); antonio.messineo@unikore.it (A.M.)

**Keywords:** waste-hydrochar, bitumen binder, rheological behavior, physical properties, aging resistance

## Abstract

In line with circular principles and the reuse of waste products, this study investigates the use of a waste-derived additive sourced from civil waste to modify the rheological and physical properties, as well as the aging resistance, of bitumen. Different dosages of waste-hydrochar (HC), produced via hydrothermal carbonization of digested sewage sludge, specifically 2%, 4%, and 10% by weight, were introduced to the bitumen, and the materials were characterized in terms of their rheological, physical, and aging behavior. Two aging protocols, e.g., short-term thermal aging and UV irradiation aging, were followed to evaluate the aging resistance of the bitumen with and without waste-hydrochar. The results obtained suggest that bitumen containing waste-hydrochar exhibits similar rheological and physical properties to bitumen without an additive, indicating the potential for using this waste material as a suitable bitumen additive. Furthermore, the presence of waste-hydrochar does not reduce the short-term thermal or UV irradiation resistance of bitumen, again suggesting the potential for using this waste material as a suitable bitumen additive. Finally, the results obtained have been compared with those of bitumen containing high-cost biochar, highlighting the potential to replace high-cost biochar with low-cost, waste-hydrochar.

## 1. Introduction

The increasing emphasis on sustainable road construction has resulted in the incorporation of secondary raw materials into asphalt mixtures. This development is driven by two primary objectives: firstly, to mitigate environmental impacts, and secondly, to enhance pavement performance.

In general, the addition of specific materials, such as elastomers/plastics or additives, to bitumen has been shown to enhance its overall performance [1,2,3]. For instance, Polymer Modified Bitumen (PMB) refers to bitumen (asphalt binder) that has been modified by adding elastomers or plastics to enhance its performance characteristics, such as elasticity, strength, temperature susceptibility, and resistance to deformation or cracking [4]. However, PMB, with improved elasticity and recovery ability, can be obtained by adding elastomers such as copolymers Styrene-Butadiene-Styrene (SBS) or Styrene-Butadiene Rubber (SBR) or rubber from recycled tires, as Crumb Rubber (CR) [5,6,7,8]. To improve stiffness and rutting resistance, PMB can be formulated by adding plastics, such as EVA (Ethylene-Vinyl Acetate), PE (PolyEthylene), and PP (PolyPropylene) [9,10].

Therefore, it has been determined that the presence of small particles exerts a substantial influence on the rheological characteristics of bitumen, a consequence of their distinctive properties, such as their elevated surface area-to-volume ratio. It has been demonstrated that meaningful alterations to the chemical composition of materials can be achieved, even when only small quantities of modifying agents are employed. In this context, a particular approach to bitumen modification that warrants greater research attention is the incorporation of the bituminous binder with additives that have the capacity to enhance its photo-oxidative resistance [6,11,12,13].

This is an aspect that requires significant attention, as oxidative aging is an inevitable phenomenon that progressively deteriorates the long-term performance of bituminous binders [14,15,16].

A interesting current study by Jin et al. proposed the use of particles from Cathode-ray tubes (CRTs), e.g., an ingredient of glass used in obsolete televisions or computer monitors, as a filler for asphalt binders. Although the CRT glass contains a considerable amount of heavy metals, and the landfilling of CRT glass is significantly harmful to the environment, the proposed recycling method through incorporation in asphalt binders can be considered as an environmentally friendly alternative, in which the optimal addition content of CRT glass powders could be up to 10% (wt.) [17].

Although some studies have been conducted on the use of biochar and other carbonaceous materials in asphalt, there are few studies on waste-hydrochar obtained from non-biomass sources [18,19,20]. In particular, char derived from municipal sludge is characterized by high carbon content, a porous structure, and good thermal stability, making it a promising additive for bituminous materials. However, it should be noted that, since it originates from municipal waste, it may also contain many impurities, including heavy metals, which must be taken into consideration [21].

One innovative method to produce carbon-rich particles, suitable for polymers and bitumen, is the Hydrothermal Carbonization (HTC). The HTC is a thermochemical process concerning the conversion of biomasses (such as agricultural waste, sewage sludge, or food waste) or waste into a carbon-rich solid product called hydrochar under hot, pressurized water conditions. More specifically, HTC is a thermochemical process that uses water under autogenous saturated pressure. It is typically carried out at temperatures between 180 and 250 °C, with corresponding self-generated pressures of 10–50 bar [22]. The ratio of feedstock to water generally ranges from 3% to 30% by weight on a dry basis, while the reaction residence time can vary from a few minutes to several hours depending on the desired degree of carbonization and the characteristics of the feedstock. The process of hydrothermal carbonization (HTC) has been shown to facilitate the breakdown of biomass through a series of reactions, including hydrolysis, decarboxylation, condensation, polymerization, and aromatization. These reactions are enabled by the presence of water under subcritical conditions. The process of synthesis yields three primary products: The solid phase is characterized by its carbon-rich nature and is denoted as hydrochar. The aqueous phase, designated as process water (PW), is characterized by its enrichment with organic compounds, including acetic, levulinic, and formic acids [23,24]. The gaseous fraction, accounting for a minor proportion, is predominantly composed of carbon dioxide. In comparison with untreated biomass, hydrochar demonstrates superior fuel properties, including increased energy density, improved dewaterability, reduced O/C ratio, lower inorganic content [25,26,27], and enhanced combustion behavior [28,29]. In contraposition to conventional dry thermochemical conversion methods, HTC offers several advantages: it operates at lower temperatures, benefits from the catalytic role of water, and achieves high conversion efficiencies while reducing overall energy consumption. This carbon-rich by-product is a promising way to make use of waste, supporting the principles of the circular economy while potentially improving the performance of road materials in terms of both function and the environment.

In this study, the valorization of carbonaceous by-products, specifically waste-hydrochar, is proposed as a novel approach, and specifically, waste-hydrochar was used as a sustainable additive for asphalt mixtures. This waste-hydrochar was obtained from the hydrothermal carbonization (HTC) of sewage sludge. Therefore, bitumen (50–70 penetration grade), including 2, 4, and 10 wt.% waste-hydrochar (HC), was prepared and fully characterized. In terms of circularity, the waste-hydrochar originates from household waste that has undergone thermal treatment to reduce its water content and produce materials that are easy to dispose of.

This study explores the potential for using the sustainable filler in bitumen to produce materials with acceptable performance characteristics. Previously, the same bitumen (50–70) was additivated with 2, 4, and 10 wt.% of a commercial biochar (C-BCH), and the results were published elsewhere [20].

Therefore, the current study proposes replacing high-cost C-BCH with low-cost HC to formulate bitumen materials with properties and performance similar to those of high-cost bitumen composites. The results obtained suggest that high-cost C-BCH can be replaced successfully with low-cost HC to formulate bitumen with acceptable properties.

## 2. Materials and Methods

### 2.1. Materials

#### 2.1.1. Bitumen

A 50–70 penetration grade bitumen, commonly used for road applications, was utilized in this study. The general properties of the bitumen were evaluated according to European standards. The penetration at 25 °C, determined according to EN 1426 [30], was 52.25 dmm, while the Ring and Ball softening point, assessed according to EN 1427 [31], was 52.6 °C. After short-term aging, as specified by EN 12607-1 [32], the penetration at 25 °C decreased to 39.3 dmm, and the Ring and Ball softening point increased to 56 °C.

The scale of the graph of the penetration test is expressed in tenths of a millimeter (0.1 mm, i.e., dmm), which is the unit of measurement used for penetration.

#### 2.1.2. The Waste-Hydrochar

The waste-hydrochar used in this study was obtained by hydrothermal carbonization (HTC) and collected from a municipal wastewater treatment plant (WWTP) in Palermo province, Italy, which uses a Conventional Activated Sludge (CAS) technology. The sewage sludge was produced via anaerobic digestion for a duration of 28 days at a temperature of 30 °C, of thickened primary sewage sludge. The sample was characterized and stored in a refrigerator at 4 °C in a sealed plastic container until use. The feedstock utilized in this study was characterized in terms of its total solids content (TS), which was measured by subjecting it to a drying process in a ventilated oven (Umbra Forni, San Martino in Campo 06132 (PG), Italy) at a temperature of 105 °C for a minimum duration of 12 h, until constant weight was reached.

### 2.2. Preparation of Bitumen with Waste-Hydrochar

The samples of waste-hydrochar bitumen (HC) were prepared by a Silverson high-shear mixer (Silverson Machines Ltd., Chesham, Buckinghamshire, United Kingdom) with a rotational speed of 3000 rpm at 180 °C for a period of 2 h. For these purposes, three different percentages of waste-hydrochar were considered: 2% wt/wt (referred to as 2% HC), 4% wt/wt (referred to as 4% HC), and 10% wt/wt (referred to as 10% HC).

### 2.3. Methods

#### 2.3.1. Hydrothermal Carbonization Procedure

Hydrothermal carbonization (HTC) reactions were carried out in a 500 mL stainless steel autoclave (Col-Int Tech High-Pressure Reactor) equipped with a 2 kW vertical electric furnace, an electrical stirrer, and an internal coil for cooling down the reactor at the end of the reaction. Reactions were carried out at a fixed temperature of 190 °C, a fixed residence time of 1 h, and using the as received feedstock (25% of TS). In order to ensure the reliability of the results, the reaction tests were conducted three times. For each experiment, approximately 350 ± 0.5 g of the as received sample was loaded into the reactor. Drying pretreatment and subsequent addition of water to sewage sludge were purposely avoided to not alter the original properties of the feedstock [33]. Before the reaction, the autoclave was flushed three times with pure nitrogen (Airliquide, Alphagaz 1) to eliminate any oxygen in the vessel, and the system heated up to the set temperature (heating time approximately 30 min, stirring at 200 rpm).

At the end of the reaction, the system was cooled down by means of circulated water fed by a chiller. Once at room temperature (25–30 °C), the reactor was vented and the gas phase collected in a graduated plexiglass™ cylinder filled with water to evaluate the produced volume and thus the gas mass yield. Solid residue, waste-hydrochar (HC), was recovered via Buchner filtration and dried in a ventilated oven at 105 °C until constant weight (approximately 4 h). Mass yields of hydrochar MYHC and mass yields of gas MYgas were calculated as the ratio of hydrochar and gas mass on a dry basis over the mass of the raw feedstock on a dry basis Equations (1) and (2), respectively.

MY_HC_= (mass_HC_/mass_raw_) × 100 (1)

MY_Gas_= (mass_gas_/mass_raw_) × 100 (2)

Mass yields of the liquid phase were evaluated by difference following Equation (3)

MY_Liq_= 100 − MY_HC_ − MY_Gas_ (3)

The energy densification ratio (EDR) and the energy yield (EY) of waste-hydrochar were determined via Equations (4) and (5), respectively:

EDR(%)= (HHV_HC db_/HHV_raw db_) × 100 (4)

EY(%)= MY_HC_ × EDR (5)

where HHV_HC,db_ and HHV_raw,db_ are the higher heating values of hydrochar and raw feedstock (on a dry basis), respectively; MY_HC_ is the hydrochar mass yield computed as the mass of dry hydrochar collected to the mass of initial dry feedstock. 

Hydrochar fuel ratio (FR) was defined as the ratio between the fixed carbon (FC) and the volatile matter (VM), determined via proximate analysis, Equation (6):

FR = FC/VM(6)

#### 2.3.2. Characterizations of Waste-Hydrochar and Bitumen with Waste-Hydrochar 

The pH of the shar was measured using an XS Instruments Bench pH-meter by placing 1 g of solids in 50 mL of deionized water, shaking for at least 60 min, allowing the mixture to settle for 10 min, and then registering the pH. 

Raw materials and corresponding hydrochars are characterized in terms of high heating values (HHV) using a LECO AC 500 Calorimeter according to the CEN/TS 14918 standard [34]; elemental analysis (CHN) using an 828-CHN LECO Elemental analyzer with argon as a carrier gas and EDTA as a calibration standard, and proximate analysis was carried out by a LECO Thermogravimetric Analyzer TGA 701 (ASTM D7582-15 standard method) [35].

Approximately 300 mg of each solid sample was used to quantify volatile matter (VM), ashes (ash), and fixed carbon (FC) as follows: 5 °C/min ramp to 105 °C in air, held until constant weight (<±0.05%) to remove moisture in open crucibles; 16 °C/min ramp from 105 to 900 °C, hold time 7 min, in nitrogen and covered crucibles to determine VM as the mass lost; natural cooling down to 500 °C in nitrogen; 30 °C/min ramp in air to 800 °C and isothermal until constant weight in open crucibles to evaluate ASH as the remaining mass. FC was estimated by subtracting VM and ash percentages from 100%. Total organic carbon (TOC) was determined by a Shimadzu TOC-VWP Analyzer (liquid samples were previously filtered through 0.45 μm syringe filters). Inductively Coupled Plasma—Optical Emission Spectroscopy, ICP-OES, (AvioTM Max 220—Perkin Elmer—Shelton, CT, USA), was used to determine the inorganic elemental concentration of the sludge and corresponding hydrochar samples. Samples were oven-dried at 105 °C until constant weight and then acid sewage sludge in concentrated nitric acid (Sigma Aldrich—70% purified by redistillation) using a single reaction chamber microwave digestion system (UltraWAVE, Milestone Inc., Sheldon, CT, USA) and Teflon-lined vials to prevent interference.

The samples of bitumen with waste-hydrochar at three different percentages were subjected to rheological analysis at different temperatures using a Brookfield viscometer (DV-III™ Ultra Rheometer, Middleboro, MA, USA), in accordance with EN 13302 [36]. In accordance with EN 14770 [37], dynamic mechanical analysis in the Linear viscoelastic range was carried out using (Anton Paar Physica MCR 10). Frequency sweep tests were carried out in a temperature range from −10 °C to 180 °C, using a plate–plate geometry. Depending on the temperature test, two different plate sizes were used: an 8 mm diameter plate for tests at lower temperatures and a 25 mm diameter plate for those at higher temperatures. Different rheological parameters were measured, including the complex shear modulus (G*), storage modulus (G′), loss modulus (G″), and phase angle (δ).

All blends investigated were subjected to two separate aging procedures. First, thermal aging was performed through the RTFOT procedure, followed by photo-oxidative aging, simulating prolonged exposure to UV radiation and oxidative environments. These procedures were conducted to assess the evolution of mechanical properties over time and under environmental stress. The photo-oxidative aging was performed using a Q-UV weathering chamber (Q-LAB, Westlake, OH, USA), equipped with UVB-313 nm fluorescent lamps. The test was conducted at a constant temperature of 70 °C, simulating prolonged exposure to ultraviolet radiation and oxidative environmental conditions.

The progression of photo-oxidative aging was monitored via spectroscopic analysis using Attenuated Total Reflectance-Fourier Transform Infrared Spectroscopy (ATR-FTIR) performed with a Spectrum One spectrometer (PerkinElmer, Waltham, MA, USA). For each sample, multiple ATR-FTIR analyses were conducted at different points until overlapping spectra were observed, ensuring reproducibility.

UV irradiation aging was investigated by examining spectral changes at various exposure intervals, with particular focus on two characteristic absorption bands: the carbonyl peak centered around 1700 cm^−1^ and the hydroxyl peak in the range of 3600–3200 cm^−1^.

## 3. Results and Discussion

### 3.1. Characterization of Waste-Hydrochar 

Table 1 report the HTC products mass yields, pH, energy properties, and elemental analysis, respectively. 

Hydrothermal carbonization at 190 °C produced a high waste-hydrochar (HC) mass yield (83.4%). The liquid fraction (15.55%) indicates moderate solubilization of organics, while gas formation remains negligible (1.05%), confirming the low-temperature, high-solid-retention nature of the process. The energetic properties of the HC reflect this limited transformation. The HHV increases only marginally (12.05 to 12.79 MJ kg^−1^), yet the energy yield remains high (88.52%), indicating that most of the feedstock’s calorific value is retained in the solid product. The energy densification ratio (106%) confirms a small improvement in energy density, although the overall enhancement is constrained by the intrinsically high ash content of the raw material. Proximate analysis supports this interpretation: the HC shows slightly lower volatile matter and higher fixed carbon, but also a notable increase in ash content (from 42.34% to 45.54%). The resulting fuel ratio increases only marginally. Elemental analysis further highlights the modest degree of carbonization at 190 °C. Carbon and hydrogen contents decrease slightly, while nitrogen increases, largely due to concentration effects resulting from mass loss during HTC. The reduction in oxygen, consistent with dehydration reactions, indicates the initial onset of HTC-driven aromatic stabilization, although these changes remain limited at this temperature.

Table 2 reports the total solid content (TS) evaluated at 105 and 550 °C, total organic carbon and inorganic element composition on a dry basis. The transformation of solids during HTC is also reflected in the total solid content and the distribution of inorganic elements. Both TS at 105 °C and 550 °C decrease in the hydrochar, indicating partial removal of moisture-bound and volatile organic matter. TOC decreases from 34.6% to 29.5%, confirming that a portion of organic carbon is transferred to the process water. In contrast, all analyzed metals (Cd, Ni, Pb, Cu, and Zn) show increased concentrations in the hydrochar, as expected due to their non-volatility and the high mass retention of the solid phase. In particular, Zn and Cu reach markedly elevated values, typical of sewage-sludge chars and potentially relevant for regulatory considerations related to land application. Mercury remains essentially unchanged, consistent with its low initial concentration and the mild reaction conditions. Overall, HTC at 190 °C results in a hydrochar that is moderately altered; thus, the process stabilizes the material and improves its energy properties. 

**Figure 1:**
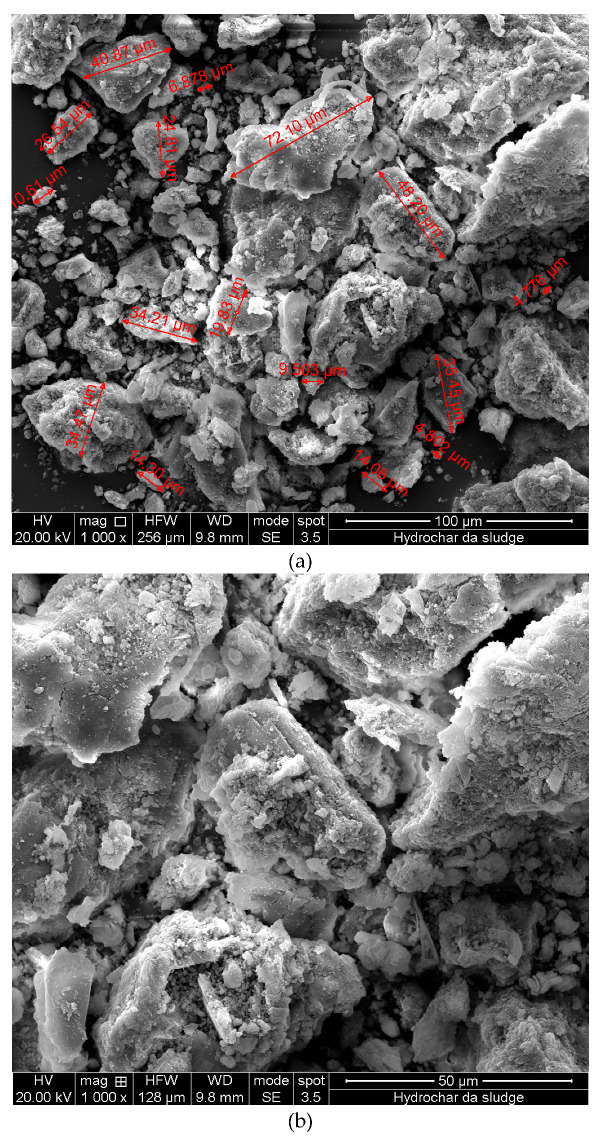

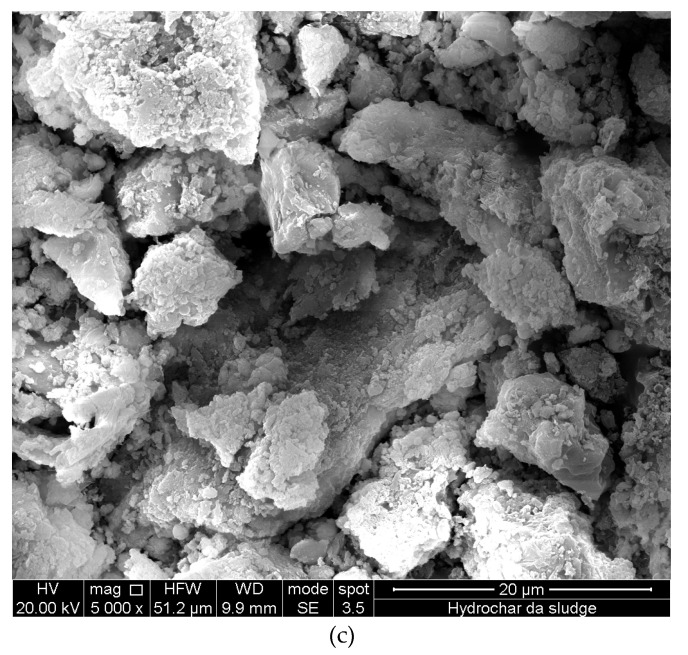
SEM micrographs of waste-hydrochar (HC) particles at different magnifications: (**a**) 100 μm; (**b**) 50 μm; (**c**) 20 μm.

Figure 1a–c shows SEM images of waste hydrochar (HC) particles at different magnifications. It is worth noting that HC powder comprises numerous particles of different sizes, shapes, and dimensions, see Figure 1a. Furthermore, in Figure 1b,c, it is clearly observed that the dimensionless sizes of HC particles are different. However, the particle size distribution is not representative because dimensions depend not only on HTC process conditions but also on factors such as waste composition and temperature. Furthermore, HC particles are extremely brittle in powder form, and their dimensions change during mixing with bitumen. 

### 3.2. Characterizations of Bitumen and Modified Binders

**Figure 2:**
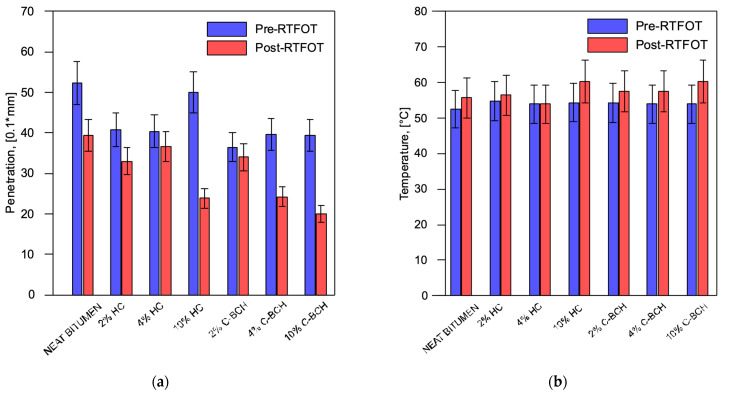
(**a**) Penetration test and (**b**) softening point of unaged and aged neat bitumen and bitumen binders containing HC and C-BCH samples. (The data of bitumen/C-BCH were published before [20]).

The results of penetration and softening point tests of all samples investigated in this study, both unaged and aged, are summarized in Figure 2a,b. The results obtained by analyzing the bitumen filled with waste-hydrochar (HC) were compared with those of bitumen filled with commercial biochar (C-BCH), all prepared under identical processing conditions. The graphs referring to penetration and softening points show that, in general, by adding HC or C-BCH, the stiffness of the bitumen increases as the percentage of filler added increases, as was expected. For each mixture investigated, three repetition tests were performed, and the average value was plotted. The data of bitumen/C-BCH were published before [19]. In addition, there is no significant difference between the addition of a commercial product (e.g., C-BCH) and the addition of a waste product (e.g., HC).

**Figure 3:**
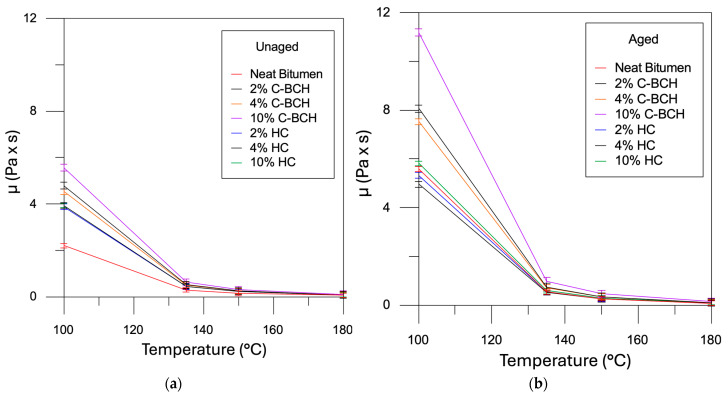
Dynamic viscosity of binder, measured with a Brookfield rotational viscometer of (**a**) unaged and (**b**) aged W-BCH and C-BCH samples. (The data on bitumen/C-BCH were published before [20]).

Dynamic viscosity measurements were carried out on all the samples, with the neat bitumen tested separately for comparison. Figure 3 presents the results of the viscosity tests conducted. The viscosity of the modified char samples, whether it is a commercial product (e.g., C-BCH) or a waste material (e.g., HC), increases as the amount of filler added increases, in accordance with the results of the empirical tests. 

Assessing the extent of this increase is crucial, as an excessive rise in viscosity can hinder the practical application of these modified binders in road construction. For this reason, the viscosity at 135 °C was measured for each sample, and the corresponding values are illustrated in Figure 3.

Viscosity at 135 °C is a crucial parameter for evaluating the processability of bituminous binders in asphalt mixture production. To ensure efficient pumpability and handling at the industrial scale, technical specifications require this value to remain below 3 Pa·s [19]. In the present study, all tested samples met this criterion, indicating their suitability for large-scale application and compliance with current sustainability-oriented production standards.

Furthermore, the two types of char exert distinct effects on bitumen viscosity; specifically, the waste-hydrochar induces a less pronounced modification than the commercial additive.

**Figure 4:**
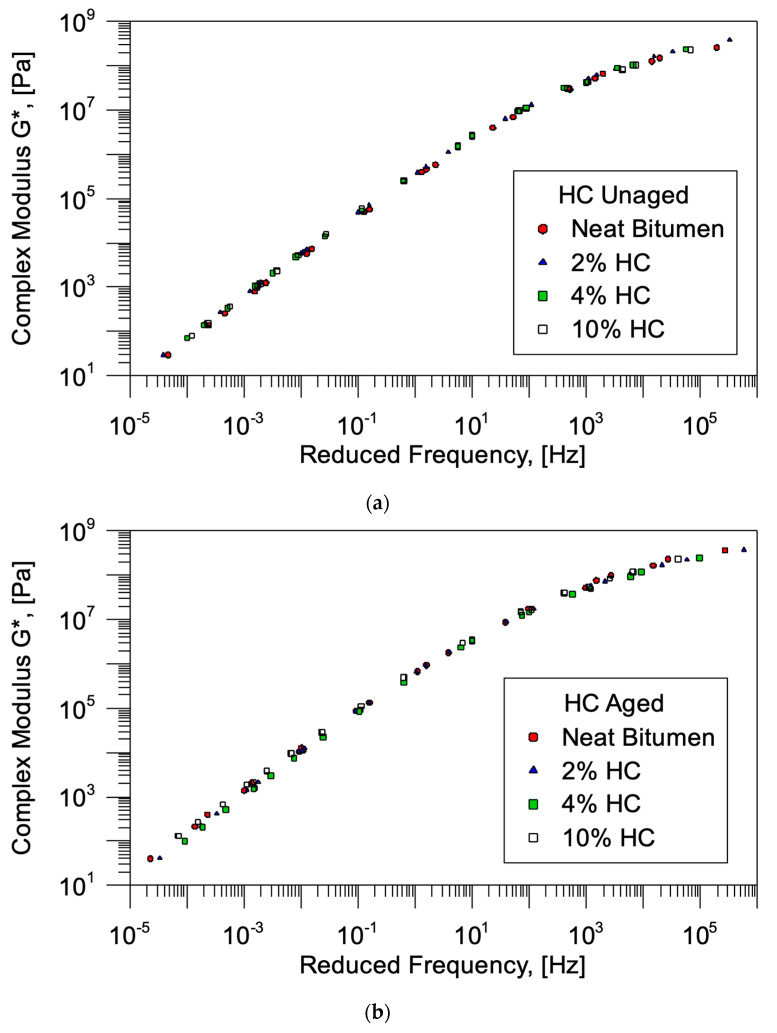
Master curve of complex modulus (G*) of (**a**) unaged and (**b**) aged W-BCH and C-BCH samples as a function of reduced frequency.

Figure 4 shows the complex modulus of all the samples investigated in this study before and after short-term aging. Prior to aging (see Figure 4a), all binders exhibit a comparable viscoelastic behavior. The shape and trend of the master curves nearly overlap, indicating that the addition of biochar does not substantially affect the rheological behavior of the unaged bitumen. Although a slight increase in stiffness is observed with increasing biochar content, the overall viscoelastic response remains comparable to that of the neat binder. This suggests that the waste-hydrochar can be incorporated without significantly compromising the processability and workability of the binder during production.

Following short-term aging (see Figure 4b), an overall increase in complex modulus was observed for all samples. This behavior is consistent with the anticipated effects of oxidation, which leads to hardening of bitumen. However, this increase is less for biochar-modified binders, particularly at higher frequencies, where the effect of oxidative stiffening is generally more evident. The presence of biochar appears to reduce the sensitivity of the material to aging, confirming its partial protective effect. 

The rheological curves display a comparable shape, exhibiting a modest increase in viscosity that remains within the acceptable limits for road applications.

Overall, the comparison between unaged and aged master curves shows that the addition of char leads to a modest increase in the initial stiffness of the binder, as well as a reduction in the aging-induced viscosity increase. These results demonstrate that the waste-hydrochar slightly enhances the oxidative stability of bitumen without altering its fundamental viscoelastic behavior.

Table 3 provides a summary of the crossover frequency and crossover modulus values of the neat, commercial, and waste-hydrochar-modified bitumen, both before and after short-term aging. It should be noted that the results concerning the C-BCH biochar were previously discussed in detail in a separate publication by the same authors [20]. The crossover parameters, determined from the intersection point of storage and loss modulus, are indicative of the transition between elastic and viscous behavior. This provides insight into the structural organization and aging sensitivity of the binders.

Prior to the aging process, both categories of filler have been observed to enhance the crossover modulus in comparison with neat bitumen, suggesting an elevated initial stiffness. The increase is particularly evident for commercial biochar (C-BCH), which shows values up to 4.57 × 10^7^ Pa at 10 wt.%, compared with 2.32 × 10^7^ Pa for the neat bitumen. Compared to the neat bitumen, in the case of the addition of waste-hydrochar, a very slight increase in the crossover point, both in terms of frequency and modulus, was observed, changing from 2.32 × 10^7^ Pa to 2.46 × 10^7^ Pa. This suggests that the commercial biochar performs a more substantial role in the development of a stiffer microstructure. The crossover frequency values further confirm this trend: the samples containing biochar generally exhibit higher crossover frequencies, which is indicative of an enhanced elastic response at higher loading conditions.

After the aging process, a decline in both the crossover frequency and modulus was evident in all samples. This aspect can be attributed to the structural changes that are correlated with the oxidation and hardening of the bitumen matrix. However, the decrease is less pronounced for the biochar-modified binders, especially those containing C-BCH, indicating enhanced structural stability and resistance to oxidative degradation. In particular, the 10% C-BCH sample exhibited a relatively high crossover modulus (3.75 × 10^7^ Pa), so confirming the protective effect of biochar against aging. Conversely, the bitumen incorporating waste-hydrochar displayed a more moderate behavior; after aging, its crossover modulus values and complex modulus values remained similar to those of neat bitumen.

**Figure 5:**
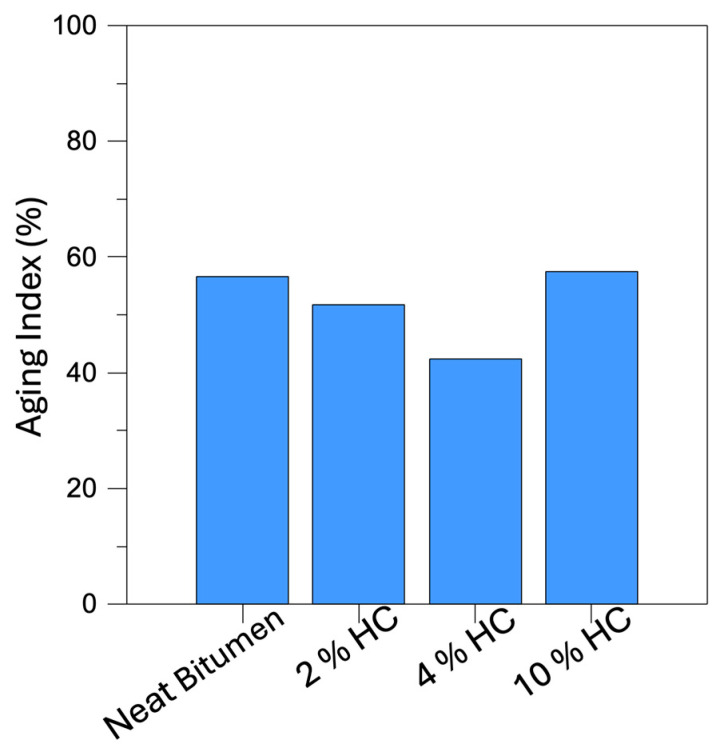
Aging Index (AI) of all samples investigated.

As shown in Figure 5, the aging index (AI) values for neat bitumen and waste-hydrochar-modified bitumen are summarized. The AI provides an indication of the material’s susceptibility to oxidative aging: lower values correspond to better resistance to aging. As demonstrated in Figure 1, the incorporation of biochar, derived from waste, generally enhances the resistance of the bitumen to aging when compared to the neat binder. The binder with 4% HC exhibits the lowest AI value, confirming an optimal balance between filler content and bitumen interaction, which likely limits oxygen diffusion and retards oxidation. The mixture containing 2% HC demonstrates a moderate enhancement, while the 10% W-BC sample exhibits a higher AI, indicating that excessive biochar content may impede homogeneity and adversely affect aging resistance. The findings of the present study demonstrate that the addition of an appropriate amount of additive can enhance the durability of bituminous binders. This suggests that waste-hydrochar is a sustainable and cost-effective alternative to conventional additives.

**Figure 6:**
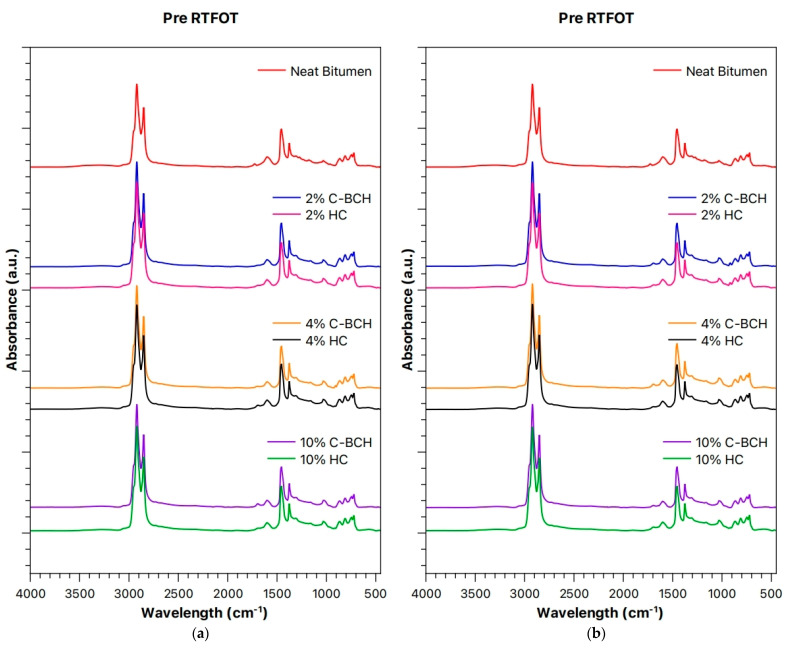
ATR-FTIR Spectra at time zero (before UV-b exposure) of (**a**) unaged and (**b**) aged HC and C-BCH samples compared to neat bitumen. The data on bitumen/C-BCH were published before [20].

Figure 6 displays the ATR-FTIR spectra of unaged (pre-RTFOT) and aged (post-RTFOT) bitumen samples with and without waste-hydrochar (HC) and commercial biochar (C-BCH). As is noticeable, both spectra of neat pre-RTFOT and neat post-RTFOT bitumen are identical, and we cannot detect any additional peak due to thermal aging, considering the heterogeneous composition of bitumen binder. Overall, the bitumen (50–70, typically used in Italy/Europe) is a complex mixture of hydrocarbons, and its SARA fractions are approximately as follows: Saturated linear and branched hydrocarbons at ~5–15%, Aromatics at ~40–60%, Resins at ~15–30%, and Asphaltenes at ~5–25%. However, as expected, the ATR-FTIR spectra of neat pre-RTFOT and neat post-RTFOT bitumen appear very complex, and the assignment of the peaks is a hard matter due to potential overlapping of SARA constituents.

Therefore, the incorporation of both HC and C-BCH does not result in the emergence of new absorption bands or the disappearance of existing ones. This observation suggests that the chemical structures of the bituminous binders remain largely unaffected by the presence and considered concentrations of these additives, i.e., HC and C-BCH, indicating the absence of significant chemical interactions between the bitumen matrix and the added chars.

**Figure 7:**
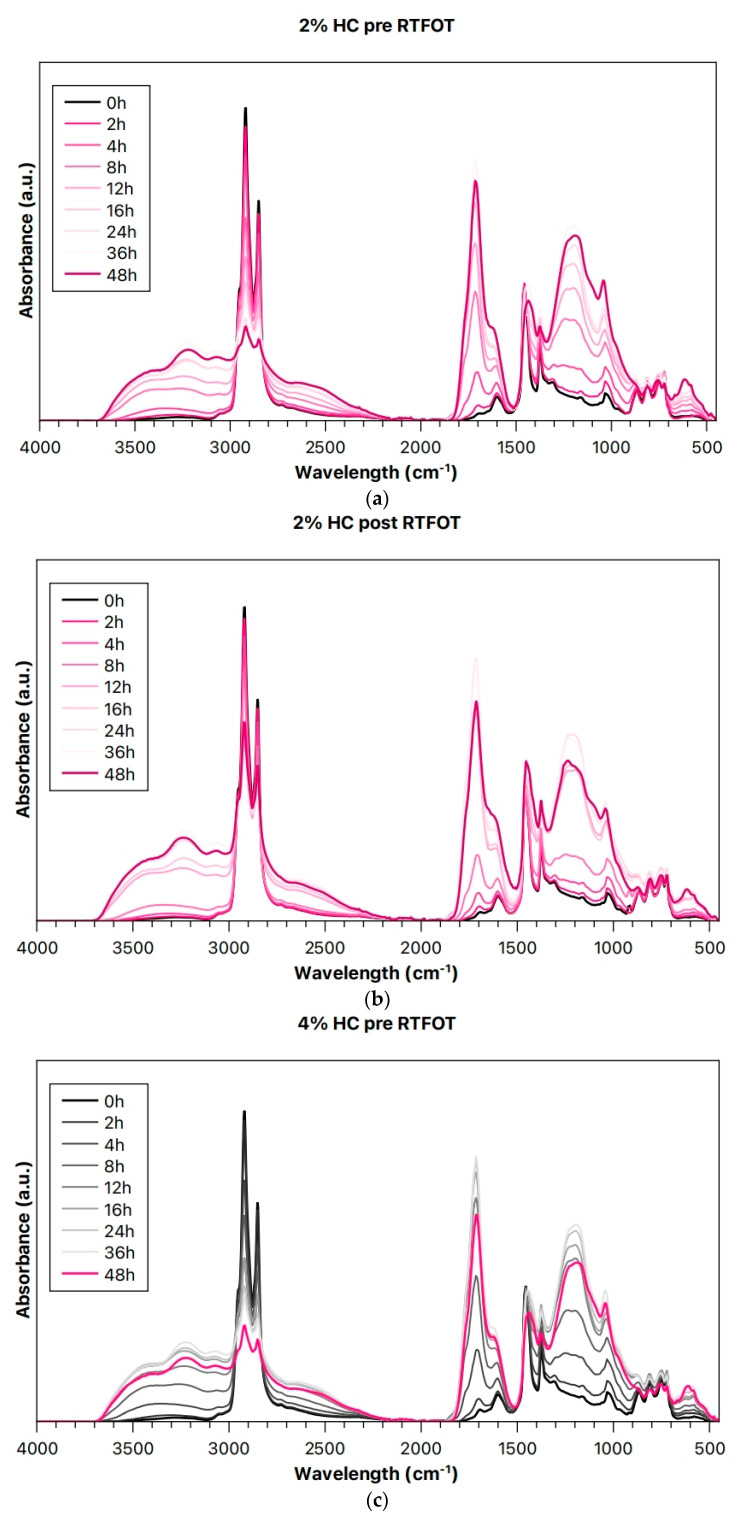

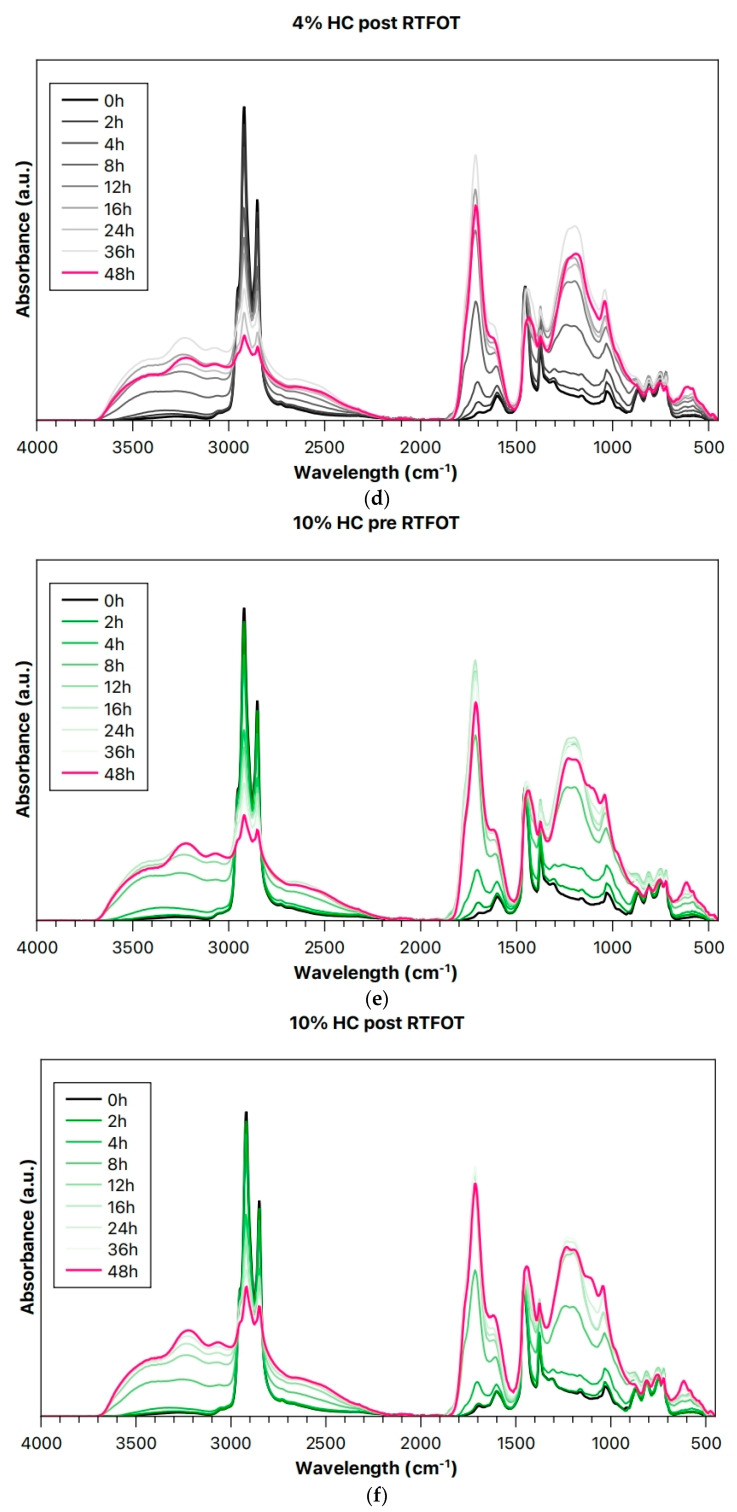
ATR-FTIR spectra of (**a**,**c**,**e**) unaged (pre-RTFOT) and (**b**,**d**,**f**) aged (post-RTFOT) bitumen binders containing 2, 4, and 10% HC as a function of UV irradiation times.

Figure 7 presents the complete ATR-FTIR spectra of unaged (pre-RTFOT) and aged (post-RTFOT) bitumen samples containing 2, 4, and 10 wt.% waste-hydrochar (HC) subjected to different durations of UV irradiation. A clear trend is observed across all samples, with a pronounced increase in the absorption intensities corresponding to hydroxyl and carbonyl functional groups, accompanied by a noticeable decrease in the aliphatic C–H stretching bands as the UV exposure time increases.

These spectral changes indicate progressive oxidation of the bitumen matrix under UV irradiation, characterized by the generation of oxygen-containing species such as hydroxyls, carbonyls, and carboxylic acids. The formation of these functional groups suggests photo-oxidative degradation involving the incorporation of oxygen into the molecular structure of the bituminous binder. In contrast, the decline in C–H stretching peak intensity likely results from chain scission reactions, in which UV-induced cleavage of aliphatic and aromatic chains leads to fragmentation of larger hydrocarbon molecules into smaller, more oxidized compounds.

The extent of these transformations is notably more pronounced in the unaged (pre-RTFOT) samples compared with the aged (post-RTFOT) ones. This difference implies that short-term thermal aging (RTFOT) enhances the structural stability of the binder, making it more resistant to subsequent photo-oxidative degradation. The reduced susceptibility of post-RTFOT samples to UV-induced chemical changes may be attributed to the prior oxidation and volatilization of the more reactive light components during the thermal aging stage, leaving behind a more stable and less UV-sensitive residue.

Overall, these observations confirm that UV irradiation promotes oxidative degradation of bitumen through the introduction of oxygenated species and the breakdown of hydrocarbon chains, while preliminary thermal aging mitigates the extent of such deterioration by stabilizing the bituminous matrix.

The incorporation of low-cost waste-hydrochar (HC) does not negatively affect the UV irradiation resistance of bitumen, indicating that it can be considered a viable additive for binder modification. Although HC does not appear to provide a pronounced protective effect against photo-oxidative degradation, the preservation of the binder’s oxidative resistance is itself a favorable outcome. Given the dual considerations of utilizing a readily available, low-cost waste material and mitigating environmental disposal issues, the unchanged aging behavior demonstrates that HC can be integrated into bitumen without compromising performance, offering both economic and sustainability benefits.

The following equations were used to calculate the Hydroxyl Index (HI) and Carboxyl Index (CI) for the quantitative analysis [38]:

(7) $$I(OH)=\frac{Area\;of\;the\;hydroxyl\;band\;centered\;between\;3600-3299\;{cm}^{-1}}{Area\;of\;the\;{CH}_{2}\;centered\;at\;ca.\;1455\;{cm}^{-1}+Area\;of\;{CH}_{3}\;band\;centered\;at\;ca.\;1376\;{cm}^{-1}}$$

(8) $$I(CO)=\frac{Area\;of\;the\;carbonyl\;band\;centered\;at\;ca.\;1700\;{cm}^{-1}}{Area\;of\;the\;{CH}_{2}\;centered\;at\;ca.\;1455\;{cm}^{-1}+Area\;of\;{CH}_{3}\;band\;centered\;at\;ca.\;1376\;{cm}^{-1}}$$

**Figure 8:**
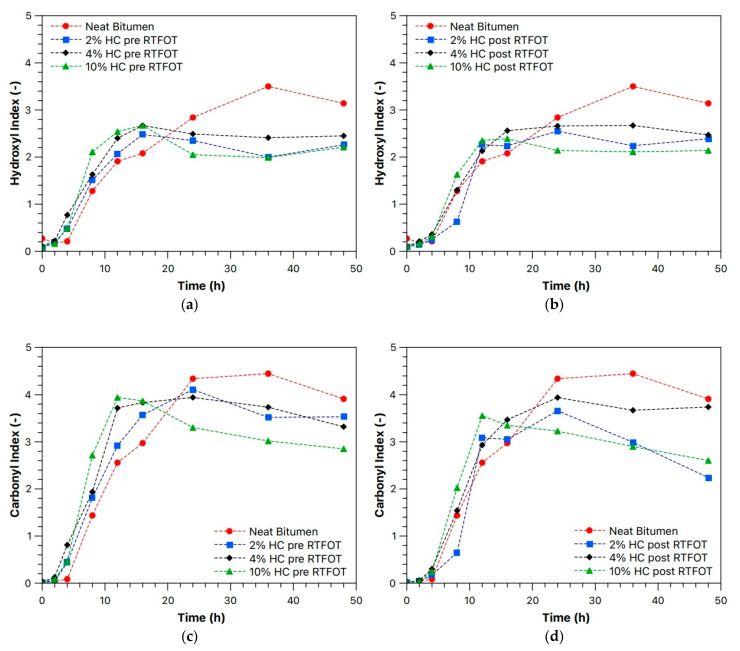
(**a**,**b**) Hydroxyl and (**c**,**d**) Carbonyl Index for HC sample as a function of waste-hydrochar concentration before (pre-RTFOT) and after (post-RTFOT) aging.

The trends of the calculated hydroxyl index (HI) and carbonyl index (CI) for the investigated samples are presented in Figure 8a–d. The neat bitumen exhibits slightly higher accumulations of hydroxyl and carbonyl groups compared to the samples modified with HC, particularly at longer UV exposure times. However, the differences between the neat and HC-modified samples are relatively small, indicating that the presence of waste-hydrochar does not substantially alter the oxidative response of the binder.

Furthermore, the HI and CI values for samples containing 2, 4, and 10 wt% HC are very similar, suggesting that the additive concentration has minimal impact on the extent of photo-oxidative degradation. These results confirm that HC does not significantly accelerate or inhibit the formation of oxygen-containing functional groups in bitumen under UV irradiation, consistent with the ATR-FTIR observations. Overall, the incorporation of HC appears to maintain the oxidative stability of the binder while allowing for the sustainable utilization of low-cost waste material.

## 4. Conclusions

The present study investigates the potential use of a sustainable additive, e.g., waste-hydrochar (HC), as a modifier for bitumen in road construction. To this end, various percentages of waste-hydrochar were incorporated into a 50–70 penetration grade bitumen. The resulting modified binders were then characterized before and after short-term aging using conventional and rheological tests to evaluate the effects of the modification.

The experimental results indicate that the addition of waste-hydrochar results in a moderate increase in the stiffness of bitumen, without compromising its workability or processability. Photo-oxidative analysis confirms that HC neither significantly accelerates nor inhibits the formation of oxygen-containing functional groups in bitumen under UV irradiation, in agreement with the ATR-FTIR observations. Overall, the incorporation of HC maintains the oxidative stability of the binder while providing a technically advantageous enhancement in stiffness, which may improve resistance to rutting under service conditions. Furthermore, the use of HC promotes the sustainable valorization of low-cost waste material, supporting both performance and environmental objectives.

Building upon these findings, the study demonstrates that such a sustainable and low-grade additive can be effectively employed in road pavement applications, achieving performance levels comparable to those previously obtained by the same authors using commercial biochar.

## Figures and Tables

**Table 1:** HTC product mass yields and pH HHV, Energy Yield (EY), Fuel Ratio (FR), proximate and elemental analysis of DS feedstock and corresponding hydrochar.

Sample	MYHC [%]	MYLiq [%]	MYGas [%]	pH	HHV_HC,db_ [MJ/kg]	EYHC [%]	EDR [%]	VM [%]	FC [%]	Ash [%]	FR	C [%]	H [%]	N [%]	O [%]
DS raw	-	-	-	7.20	12.05	-	-	49.62	8.04	42.34	0.16	40.25	4.87	0.86	11.68
HC	83.40	15.55	1.05	7.80	12.79	88.52	106.10	44.78	9.68	45.54	0.22	38.37	4.66	1.18	10.26

**Table 2:** Total solid content (TS) evaluated at 105 and 550 °C, and total organic carbon and inorganic element composition on a dry basis.

Sample	TS 105 °C [%]	TS 550 °C [%]	TOC [%] s.s.	Cd [ppm]	Ni [ppm]	Hg [ppm]	Pb [ppm]	Cu [ppm]	Zn [ppm]	P [%] s.s.
DS raw	42.1	55.5	34.6	6.7	101	0.95	83	578	1549	2.8
HC	36.2	49.4	29.5	8.7	117	0.97	99	730	1800	3.6

s.s.: suspended solid.

**Table 3:** Crossover frequency and Crossover Modulus of all investigated samples. (The data on bitumen/C-BCH were published before [20]).

Samples	Crossover Frequency, [Hz]	Crossover Modulus, [Pa]	Crossover Frequency, [Hz]	**Crossover Modulus, [Pa]**
	Unaged		Aged	
Neat Bitumen	566	2.32 × 10^7^	246	1.97 × 10^7^
2% HC	700	2.6 × 10^7^	337	1.97 × 10^7^
4% HC	632	2.40 × 10^7^	365	1.97 × 10^7^
10% HC	668	2.46 × 10^7^	285	2.12 × 10^7^
2% C-BCH	348	2.30 × 10^7^	146	1.53 × 10^7^
4% C-BCH	1525	2.64 × 10^7^	525	2.28 × 10^7^
10% C-BCH	1044	4.57 × 10^7^	380	3.75 × 10^7^

## Data Availability

The original contributions presented in this study are included in the article. Further inquiries can be addressed to the corresponding authors.
